# Adaptive Robust EKF with NARX-Based Velocity Prediction for High Precision AUV Navigation Under DVL Outages

**DOI:** 10.3390/s26134240

**Published:** 2026-07-03

**Authors:** Yuxuan Fan, Xinhui Zhang, Wenfeng Nie, Wenhao Lu, Yangfan Liu, Yubo Li, Jiandi Feng, Baomin Han

**Affiliations:** 1 School of Civil Engineering and Geomatics, Shandong University of Technology, Zibo 255049, China; 23110901027@stumail.sdut.edu.cn (Y.F.); fengjiandi2007@163.com (J.F.); hanbaomin@aitech.edu.cn (B.H.); 2 School of Space Science and Technology, Shandong University, Weihai 264209, China; wenfengnie@sdu.edu.cn (W.N.); xin2ele@163.com (W.L.); liuyangfan1995@163.com (Y.L.); 202518087@mail.sdu.edu.cn (Y.L.); 3 School of Navigation and Internet of Things, Aerospace Information Technology University, Jinan 250014, China

**Keywords:** SINS/DVL/PS underwater integrated navigation, propeller rotational speed, Sage–Husa, Huber function, NARX velocity prediction model

## Abstract

**Highlights:**

**What are the main findings?**

- In the SINS/DVL/PS integrated navigation system, an AHR-EKF that effectively combines Huber robust estimation with Sage–Husa adaptive filtering through a specifically designed mechanism is proposed. Simulation and sea trial results demonstrate that, compared with baseline methods, the proposed approach achieves significant improvements in positioning accuracy.
- A velocity prediction model based on NARX is constructed. Both simulation and sea trial results show that this method provides high-accuracy short-term velocity estimates during DVL outages, effectively suppressing the rapid accumulation of INS errors.

**What are the implications of the main findings?**

- The AHR-EKF can suppress outliers and adapt to time-varying noise, providing a new theoretical pathway for multi-source fusion navigation in complex environments.
- The proposed NARX-based velocity prediction model achieves high-precision prediction using only existing AUV sensors without relying on high-cost equipment, making it particularly suitable for harsh acoustic environments such as deep sea and under-ice conditions.
- This method significantly improves AUV navigation performance under complex seabed terrain, strong acoustic interference, and DVL outages, and can be widely applied to high-precision, long-duration missions, including deep sea exploration, pipeline inspection, military reconnaissance, and environmental monitoring.

**Abstract:**

Autonomous Underwater Vehicles (AUVs) are widely employed for deep sea exploration and underwater operations, but their navigation performance is often degraded in complex environments due to time-varying measurement noise, abnormal observations, and Doppler Velocity Log (DVL) outages. To address these challenges, this paper proposes an integrated SINS/DVL/PS navigation framework that combines an Adaptive Huber and Sage–Husa Extended Kalman Filter (AHR-EKF) with a Nonlinear AutoRegressive with eXogenous inputs (NARX)-based velocity prediction model. The AHR-EKF effectively suppresses outliers and adapts to time-varying noise, thereby enhancing filter stability and state estimation accuracy. During DVL outages, the NARX model predicts short-term AUV velocity using propeller speed, velocity increments from the navigation system, and attitude information as exogenous inputs. This data-driven approach compensates for lag and mismatch in propeller-based velocity measurements, while capturing both short-term fluctuations and overall velocity trends. Simulations and sea trials were conducted to validate the method. In the simulation experiment during DVL outages, the V-NARX method achieved east and north positioning of RMS errors of 8.397 m and 6.530 m, compared with 24.699 m and 10.218 m for the V-RPM method. In the sea trial, the V-NARX method achieved east and north RMS errors of 41.160 m and 28.023 m, respectively, compared with 52.820 m and 67.057 m for V-RPM, corresponding to reductions of 22.1% and 58.2%. The proposed method maintains trajectory continuity and effectively suppresses rapid INS error accumulation during DVL outages, significantly enhancing emergency navigation capability under DVL outages. Although its positioning accuracy does not match that of normal DVL operation, the method provides a practical and reliable engineering solution for continuous AUV navigation when DVL is unavailable.

## 1. Introduction

With the continuous development of marine resource exploitation, ocean environmental monitoring, deep sea scientific exploration, and subsea infrastructure maintenance, the ocean has become an important domain for resource utilization, scientific research, and strategic expansion [1,2]. In this context, Autonomous Underwater Vehicles (AUVs) have become essential platforms for ocean exploration and underwater operations, as they can operate in complex underwater environments and perform long-duration and high-risk missions. They play a significant role in marine geoscience surveys, environmental monitoring, seabed mapping, and target inspection [3].

In recent years, with advances in platform design, environmental perception, mission planning, and cooperative operation technologies, the application of AUVs has evolved from single-task surveys to highly autonomous, multifunctional, and refined operations [4]. Meanwhile, emerging tasks such as near-bottom high-resolution observation, deep sea precision mapping, and long-term autonomous operation in complex environments have imposed higher requirements on the maneuverability, stability, and environmental adaptability of underwater platforms [5]. Therefore, providing continuous, stable, and high-precision navigation and positioning for AUVs has become a fundamental challenge that must be addressed to accomplish complex ocean missions [6].

For AUVs, navigation and positioning capability is the foundation for path planning, attitude control, payload operation, and mission decision-making [7]. As application scenarios expand from conventional marine surveys to deep sea exploration, near-bottom observation, precision mapping, and long-term autonomous operation, the requirements for navigation systems in terms of accuracy, continuity, and reliability continue to increase [8,9]. In tasks such as track following, terrain reconstruction, target inspection, and docking recovery, navigation errors not only affect positioning results but also influence trajectory control, data spatial registration, and operational safety, thereby impacting overall mission performance [10]. This issue becomes more critical in deep sea environments, where external positioning information is scarce, environmental disturbances are complex, and mission durations are long. Once navigation results exhibit significant drift or interruption, the autonomous capability of the platform is severely affected [11]. Therefore, high-precision underwater navigation is a prerequisite for AUVs to accomplish complex ocean missions and remains a fundamental problem in integrated navigation research.

Since GNSS signals cannot effectively propagate underwater, AUVs cannot obtain continuous and stable absolute positioning references. As a result, high-precision underwater navigation mainly relies on onboard sensor integration [12]. Existing studies typically adopt inertial navigation systems (INSs) as the core of integrated navigation, due to their strong autonomy, high update rate, and good short-term accuracy [13]. However, the position and velocity estimates of INSs are obtained by integrating sensor outputs, and the errors accumulate over time. Therefore, INSs alone cannot meet the requirements of long-duration missions. To address this issue, Doppler Velocity Logs (DVLs) are commonly introduced to provide velocity observations, which help suppress error divergence and improve navigation accuracy [14]. Nevertheless, the performance of DVL measurements is sensitive to bottom-tracking conditions, seabed topography, and beam status. In complex environments, measurement degradation or even failure may occur [15]. Studies have shown that when the number of effective beams is insufficient, traditional SINS/DVL integration cannot maintain complete velocity updates, leading to significant performance degradation. In contrast, a pressure sensor (PS) can provide stable depth information and offer continuous vertical constraints. With the inclusion of PS, system observability is improved, and vertical state estimation becomes more reliable [16]. Therefore, the SINS/DVL/PS integration framework, which combines INS autonomy with DVL velocity constraints and PS depth constraints, is more suitable as a fundamental navigation architecture in complex underwater environments.

**Figure 1:**
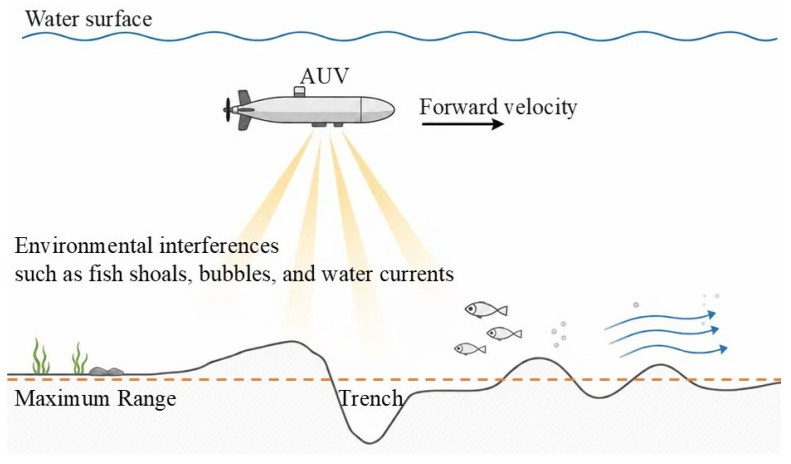
Illustration of DVL observation outages.

As shown in Figure 1, in practical applications, DVL outages are often caused by bottom-tracking failure, seabed terrain variations, rapid attitude changes, and environmental disturbances [17]. Cohen et al. pointed out that when the number of effective beams is insufficient, traditional SINS/DVL integration cannot maintain complete three-dimensional velocity updates, and system errors accumulate rapidly [18]. Sørensen et al. further showed that once key velocity constraints degrade, positioning performance deteriorates significantly [19]. Zhu et al. conducted compensation studies for DVL signal outages and demonstrated that constructing continuous and effective auxiliary constraints under DVL interruption is crucial for improving integrated navigation performance [20]. Therefore, providing continuous and reliable velocity constraints during DVL outages has become a key issue in SINS/DVL integrated navigation.

In this paper, propeller rotational speed (RPM) is selected as an auxiliary information source during DVL outages. Previous studies have shown that incorporating propulsion models and propeller speed measurements can provide effective constraints for velocity estimation [21]. However, the velocity inferred from the RPM exhibits lag and deviation from the actual AUV velocity [22]. This discrepancy mainly arises from the dynamic response characteristics of the propulsion system, hydrodynamic resistance, inertia, and environmental disturbances, which make it difficult for traditional physics-based models to fully capture the AUV’s velocity variations. In recent years, data-driven methods have been shown to effectively model such nonlinear relationships and compensate for prediction errors, particularly time series prediction models based on machine learning [23,24]. Building on this, this study develops a Nonlinear AutoRegressive with eXogenous inputs (NARX)-based model that automatically learns the system’s dynamic characteristics from historical velocity and exogenous input data, enabling high-precision prediction of the current and short-term future velocity, thereby maintaining the stability and continuity of the navigation system during DVL outages or sensor disturbances. It should be noted that the NARX model is used for short-term velocity prediction in this paper. Its lightweight structure ensures the feasibility of real-time implementation. Together with the subsequent adaptive robust filtering, it forms a prediction–filtering collaborative framework. The integration strategy between the two constitutes an important part of the proposed method.

In SINS/DVL integrated navigation, the extended Kalman filter (EKF) and its variants are widely used due to their clear structure and moderate computational cost [25]. Allotta et al. applied the unscented Kalman filter (UKF) to underwater navigation to improve the handling of nonlinearities [26]. Jin et al. proposed an adaptive nonlinear Kalman filtering method to improve state estimation performance under complex conditions [27]. To address time-varying measurement noise and the non-positive definiteness issue of covariance matrices in traditional adaptive filtering, Zhu et al. proposed an improved Sage–Husa adaptive filtering method, which enhances noise estimation and navigation accuracy in SINS/DVL systems [28]. Ma et al. further studied state estimation under the coexistence of correlated noise and outliers, and introduced an event-triggered mechanism to detect and suppress abnormal measurements, improving both accuracy and robustness [29]. In addition, Zhu et al. combined support vector regression with variational Bayesian adaptive filtering to introduce machine learning-based measurement correction, reducing the impact of abnormal observations [30]. As shown in Table 1, different methods have achieved good performance in single dimensions, such as robust outlier rejection, noise adaptation, and data-driven prediction. Existing methods can generally be categorized into two types based on their optimization mechanisms. One focuses on suppressing abnormal observations, while the other focuses on adapting to time-varying noise. M-estimation methods have strong advantages in handling outliers and abnormal observations, as they effectively reduce the influence of outliers on state estimation [31,32]. However, they mainly focus on outlier suppression and have limited capability in adapting to time-varying noise [33]. In contrast, the Sage–Husa adaptive filtering method can estimate and update noise statistics online, making it well suited for handling time-varying noise [34]. Nevertheless, it is less sensitive to large outliers and may suffer from non-positive definite covariance matrices during adaptation, which can degrade filtering accuracy and stability [32,35]. Therefore, M-estimation and Sage–Husa methods are complementary, with strengths in outlier suppression and adaptive noise estimation. Moreover, during DVL outages, multiple issues often occur simultaneously, including loss of velocity observations, time-varying noise statistics, and residual abnormal measurements. A single mechanism is insufficient to address all these problems at the same time [28,29,30].

Therefore, how to address these issues collaboratively within a unified framework remains an open technical challenge. To solve the above problems, this paper proposes an adaptive Huber–Sage–Husa extended Kalman filter (AHR-EKF). Its core idea is to exploit the complementary nature of Huber robust estimation and Sage–Husa adaptive filtering. The former suppresses the influence of abnormal observations on state estimation through weight adjustment. The latter adapts to time-varying noise by estimating the noise covariance online. The two mechanisms are coordinated through a conditional triggering strategy. The noise covariance is updated only when the residual is normal, which prevents outliers from contaminating the noise estimate. This addresses the difficulty of balancing robustness and adaptability in existing methods. During DVL outages, the NARX model provides short-term velocity predictions as observation inputs to the filter. The AHR-EKF then performs the filtering update based on these predictions. Together, they form a collaborative framework of data-driven prediction and adaptive robust filtering. Compared with existing machine learning-based methods, the NARX model offers a simpler structure and faster convergence via the Levenberg–Marquardt algorithm, making it more suitable for embedded real-time deployment.

The main contributions of this paper are summarized as follows:

(1) An EKF model combining Sage–Husa adaptive filtering and Huber robust Kalman filtering is proposed and applied to the SINS/DVL/PS integrated navigation system.
(2) During DVL outages, propeller speed information is converted into velocity constraints. This enables the system to maintain stable operation and improves state estimation accuracy under invalid measurement conditions.
(3) A NARX-based velocity prediction model is introduced to handle DVL outages. By using propeller-speed-derived velocity, navigation system velocity increments, and SINS attitude information as exogenous inputs, the model provides accurate short-term velocity estimates and preserves trajectory continuity when DVL data are unavailable.
(4) Rigorous ablation experiments are designed. Both simulation and field-test results verify the effectiveness of the proposed method and demonstrate its high accuracy and robustness in complex underwater environments.

The remainder of this paper is organized as follows. Section 1 reviews related work on underwater navigation systems and multi-sensor fusion. Section 2 introduces the fundamentals of the proposed AHR-EKF model and its integration with the SINS/DVL/PS system. Section 3 introduces a NARX-based velocity prediction method to handle DVL outages. Section 4 reports the experimental validation of the proposed method using both simulation and field-test data. Finally, Section 5 concludes the paper and discusses future research directions.

## 2. Proposed Adaptive Robust Model Based on SINS/DVL/PS

### 2.1. Measurement Equations of SINS/DVL/PS

**Figure 2:**
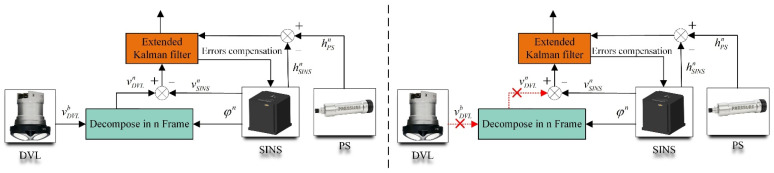
Block diagram of the SINS/DVL/PS integrated navigation system in normal mode and DVL outage mode.

In integrated navigation, SINS provides continuous state estimation. However, due to the accumulation of inertial sensor errors over time, the estimated system state gradually deviates from the true value. As shown in Figure 2, to prevent continuous error divergence, a DVL and a PS are introduced as auxiliary information to correct the system state. However, DVL measurements are often interrupted due to environmental conditions. The SINS/DVL/PS integrated model provides the state-space basis for the subsequent filtering estimation. The mathematical description and implementation details of this model are given as follows.

The state equation of the SINS/DVL/PS integrated navigation system can be expressed as:

(1) $$\dot{X}=FX+GW$$

where X is the system state vector. $\dot{X}$ is the time derivative of the state vector, representing the variation in the system state. F is the state transition matrix, which describes the dynamic evolution of the system state. G is the noise transition matrix, which relates the process noise to the system state. W is the process noise, representing random disturbances in the system.

To correct the state estimate using observation data, the integrated navigation system compares the sensor measurements with the current state through the measurement equation. The measurement equation is expressed as:

(2) Z=HX+V

where Z is the measurement vector, which includes observations from the DVL and PS. H is the measurement matrix, which describes the relationship between the state vector and the measurements. V is the measurement noise, representing errors that may occur during the measurement process.

The state vector X consists of the following components:

(3) $$X={\left[\;\;\begin{array}{ccccccccccccccc} \phi_{x} & \phi_{y} & \phi_{z} & \delta V_{E} & \delta V_{N} & \delta V_{U} & \delta P_{E} & \delta P_{N} & \delta P_{U} & \varepsilon_{x}\; & \varepsilon_{y}\; & \varepsilon_{z}\; & \nabla x & \nabla y & \nabla z \end{array}\right]}^{T}$$

where $\left[\begin{array}{ccc} \phi_{x} & \phi_{y} & \phi_{z} \end{array}\right]$ denotes the SINS attitude error, $\left[\begin{array}{ccc} \delta V_{E} & \delta V_{N} & \delta V_{U} \end{array}\right]$ denotes the SINS velocity error, $\left[\begin{array}{ccc} \delta P_{E} & \delta P_{N} & \delta P_{U} \end{array}\right]$ denotes the SINS position error, $\left[\begin{array}{ccc} \varepsilon_{x} & \varepsilon_{y} & \varepsilon_{z} \end{array}\right]$ denotes the gyroscope bias error, and $\left[\begin{array}{ccc} \nabla x & \nabla y & \nabla z \end{array}\right]$ denotes the accelerometer bias error;

(4) $$Z=\left[\begin{array}{c} Z_{DVL}\\ Z_{PS} \end{array}\right]=\left[\begin{array}{c} V_{SINS}^{n}-(I-\phi \times )C_{b}^{n}V_{DVL}^{b}\\ h_{SINS}^{n}-h_{PS}^{n} \end{array}\right]$$

(5) $$H=\left[\begin{array}{c} H_{DVL}\\ H_{PS} \end{array}\right]=\left[\begin{array}{ccccc} 0_{3\times 3} & I_{3\times 3} & 0_{3\times 3} & -C_{b}^{n}[\delta l_{DVL}^{b}]\times & 0_{3\times 3}\\ M_{pv}\;[C_{b}^{n}\;\delta l_{PS}^{b}\;]\times \; & 0_{3\times 5} & I_{3\times 1} & 0_{3\times 3} & 0_{3\times 3} \end{array}\right]$$

where $Z_{DVL}$ and $Z_{PS}$ are the velocity and depth measurements, respectively. $V_{SINS}^{n}$ is the velocity calculated by SINS, and $V_{DVL}^{b}$ is the velocity measured by DVL. $C_{b}^{n}$ is the attitude transformation matrix, and ϕ is the attitude error angle. $h_{SINS}^{n}$ is the depth calculated by SINS, while $h_{PS}^{n}$ is the depth measured and converted by the PS. $H_{DVL}$ and $H_{PS}$ are the corresponding Jacobian matrices. $\delta l_{DVL}^{b}$ and $\delta l_{PS}^{b}$ denote the lever arm errors.

### 2.2. Extended Kalman Filter

Based on the above SINS/DVL/PS integrated navigation state-space model, after discretizing the system equations, the Kalman filter can be used for recursive state estimation.

In brief, the Kalman filter process can be summarized into two core steps: prediction and update. Based on the state estimate ${\hat{X}}_{k-1}$ and the error covariance matrix ${\hat{P}}_{k-1}$ at the previous epoch, the state vector and covariance matrix at the current epoch are predicted:

(6) $$\left\{\begin{array}{c} X_{k}^{-}=\Phi_{k|k-1}{\hat{X}}_{k-1}\;\\ P_{k}^{-}=\Phi_{k|k-1}{\hat{P}}_{k-1}\Phi_{k|k-1}^{T}+Q_{k-1} \end{array}\right.$$

where $X_{k}^{-}$ is the predicted state vector at time k, $P_{k}^{-}$ is the predicted error covariance matrix, $\Phi_{k|k-1}$ is the state transition matrix, and $Q_{k-1}$ is the process noise covariance matrix.

Then, the predicted state is corrected using the measurement at the current time step, $Z_{k}$:

(7) $$\left\{\begin{array}{c} V_{k}=Z_{k}-H_{k}X_{k}^{-}\\ K_{k}=P_{k}^{-}H_{k}^{T}{\left(H_{k}P_{k}^{-}H_{k}^{T}+{\bar{R}}_{k}\right)}^{-1}\\ {\hat{X}}_{k}=X_{k}^{-}+K_{k}(Z_{k}-H_{k}X_{k}^{-})\\ {\hat{P}}_{k}=(I-K_{k}H_{k})P_{k}^{-} \end{array}\right.$$

where $V_{k}$ is the innovation vector, which represents the difference between the measurement information and the predicted information. $H_{k}$ is the measurement matrix, and $K_{k}$ is the Kalman gain matrix, which determines the weight of the measurement information in correcting the state estimate. ${\hat{P}}_{k}$ is the updated estimation error covariance matrix, and I is the identity matrix.

Through the above process, the standard Kalman filter performs recursive state estimation by state prediction, covariance prediction, innovation calculation, Kalman gain calculation, and measurement update.

### 2.3. Huber Robust Kalman Filter

To enhance the robustness of the system against outliers, the Huber robust weighting function is introduced to dynamically adjust the measurement noise. The Huber function is a commonly used robust weighting function, which reduces the influence of large residuals on the filter estimation.

First, the current measurement residual is normalized. The normalized residual $u_{k}$ is defined as the ratio between the residual and the standard deviation of the measurement noise:

(8) $$u_{k}=\frac{\left|V_{k}\right|}{\sqrt{\mathrm{diag}({\bar{R}}_{k})}}$$

Based on the magnitude of the normalized residual, the weight $p_{k}$ is adjusted dynamically. The Huber weighting function is used to reduce the influence of large residuals on the estimation. Specifically, the weight is defined as:

(9) $$p_{k}=\left\{\begin{array}{ll} \;\;1 & u_{k}\leq \lambda \\ k/u_{k} & u_{k}>\lambda \end{array}\right.$$

where λ is the threshold that determines the range of weight adjustment.

Based on the computed Huber weights $p_{k}$, the measurement noise covariance matrix $R_{k}$ is updated as:

(10) $$R_{k}={\bar{R}}_{k}/p_{k}$$

### 2.4. Sage–Husa Adaptive Kalman Filter

The Sage–Husa adaptive filter is an improved Kalman filtering method that can estimate measurement noise online. It is particularly suitable for systems where the noise statistics are unknown or time-varying in practice. By updating the measurement noise covariance matrix $R_{k}$ during state estimation, the method improves the adaptability to noise variations and abnormal measurements.

In the Sage–Husa adaptive filter, the measurement noise covariance matrix ${\tilde{R}}_{k}$ is updated as follows:

(11) $${\tilde{R}}_{k}=V_{k}V_{k}^{T}-H_{k}P_{k}^{-}H_{k}^{T}$$

To prevent the estimated noise covariance from becoming too small or too large, the measurement noise covariance matrix ${\tilde{R}}_{k}$ is constrained. The constraint is defined as:

(12) $$R_{k}=\left\{\begin{array}{ll} R_{k}^{\min} & {\tilde{R}}_{k}>R_{k}^{\min}\\ (1-\beta_{k^{\prime }})R_{k}+\beta_{k^{\prime }}{\tilde{R}}_{k} & R_{k}^{\min}<{\tilde{R}}_{k}<R_{k}^{\max}\\ R_{k}^{\max} & {\tilde{R}}_{k}\geq R_{k}^{\max} \end{array}\right.$$

where $R_{k}^{\min}$ and $R_{k}^{\max}$ are the lower and upper bounds of the measurement noise covariance matrix, respectively, to prevent the noise estimate from becoming too small or too large and causing filter instability.

The weighting coefficient $\beta_{k^{\prime }}$ is updated as follows:

(13) $$\beta_{k+1^{\prime }}=\frac{\beta_{k^{\prime }}}{\beta_{k^{\prime }}+b}$$

where b is the forgetting factor, typically set to a value close to 1, such as 0.9 or 0.999. A smaller value of b allows the system to adapt more quickly to recent noise variations, but an excessively small value may lead to large fluctuations in the noise estimation.

### 2.5. Proposed AHR-EKF

The AHR-EKF enhances the robustness of the Kalman filter by combining the advantages of the Huber robust weighting function and the Sage–Husa adaptive filter. It improves the ability to handle outliers and time-varying noise. The method computes the innovation and the normalized residual. The measurement noise covariance matrix $R_{k}$ is then adjusted dynamically according to the residual magnitude.

**Figure 3:**
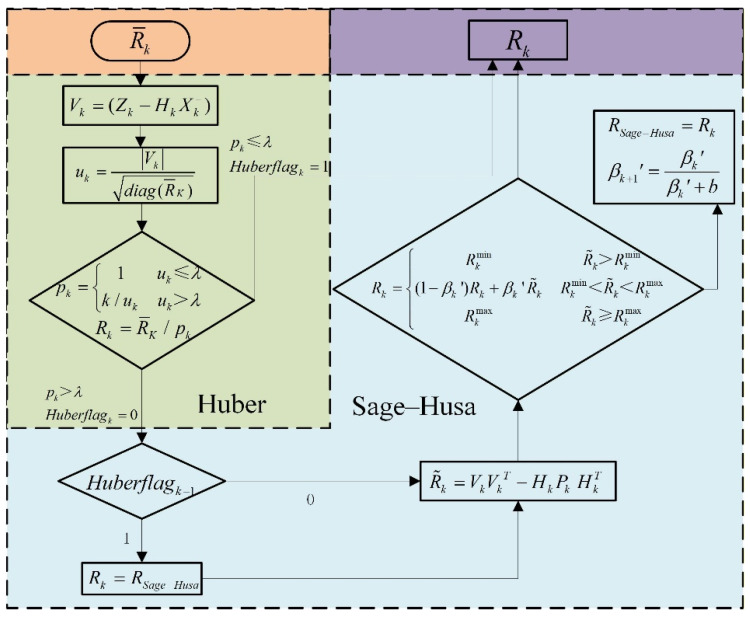
Flowchart of the proposed AHR-EKF.

The detailed procedure of the proposed method is illustrated in Figure 3 and summarized in Table 2 below.

## 3. NARX-Based Velocity Prediction Model

**Figure 4:**
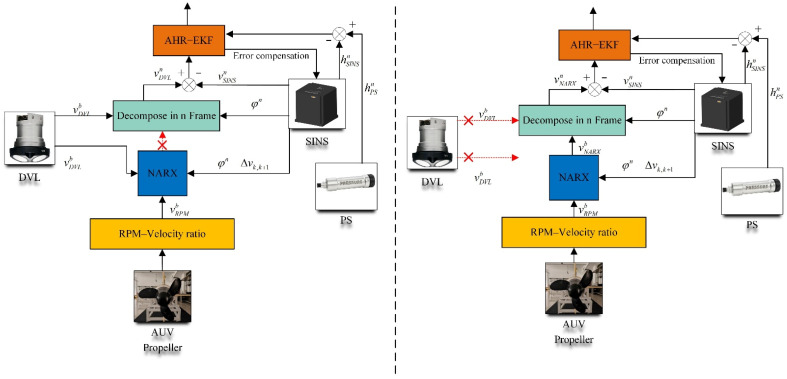
Block diagram of the proposed AHR-EKF integrated navigation system with NARX-based velocity prediction in normal mode and under DVL outages.

Figure 4 presents the complete workflow of the proposed system. The system is built upon the previous AHR-EKF framework, with additional mechanisms to handle DVL outages. In underwater environments, DVL observations are easily affected by factors such as poor acoustic reflection from soft or uneven seabeds, turbidity, or suspended particles that scatter or absorb acoustic signals. These factors can cause temporary or prolonged loss of velocity measurements, which must be considered in the design of integrated navigation systems. To address this issue, a velocity prediction model based on the NARX neural network is proposed.

### 3.1. Principles of NARX

The NARX model is a type of neural network commonly used for time series prediction and dynamic system modeling. Unlike conventional linear autoregressive models, NARX can capture the nonlinear characteristics of the system while simultaneously accounting for the influence of exogenous inputs on system outputs.

**Figure 5:**
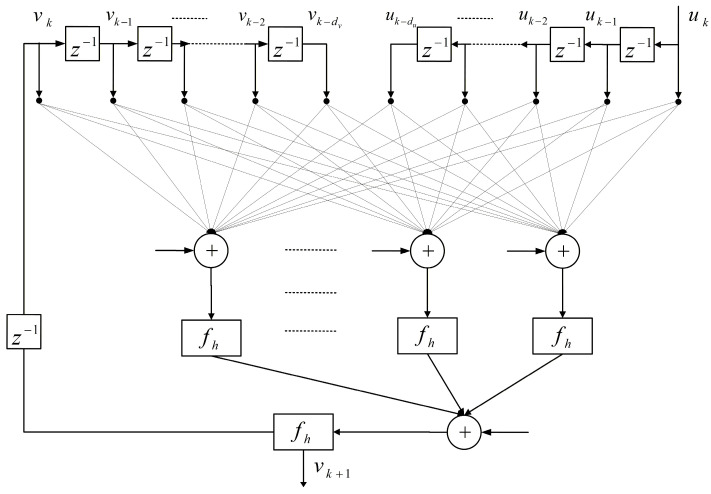
Schematic diagram of the NARX-based velocity prediction model.

As illustrated in the schematic diagram of the NARX model in Figure 5, its core idea is to use historical output and input sequences to predict the current output. Its general form can be expressed as:

(14) $$v_{k+1}=f_{h}\left(v_{k},v_{k-1},\ldots ,v_{k-d_{v}},u_{k},u_{k-1},\ldots ,u_{k-d_{u}}\right)+e_{k}$$

where $v_{k+1}$ is the system output at the current time step, $f_{h}$ is a nonlinear function approximated by a radial basis function network or a multilayer perceptron, $u_{k}$ denotes the exogenous input sequence, and $e_{k}$ represents a white noise term or modeling error. $d_{v}$ and $d_{u}$ are the output and input delay orders, respectively.

The NARX model can form a closed-loop prediction mode through its dynamic feedback mechanism, enabling multi-step forecasting and free-running system prediction.

The reasons for selecting NARX as the training model in this study are as follows. First, NARX employs feedforward neural networks to fit the complex nonlinear relationships in underwater sensor data. Second, the model can handle multiple exogenous inputs, such as propeller speed, IMU attitude, and velocity increments, reducing dependence on DVL velocity measurements. Third, the delay structure allows the model to capture the influence of historical velocity states on the current output. Finally, NARX supports multi-step prediction; when DVL measurements are unavailable, the model can directly switch to closed-loop mode, using the previous predicted output as the input for the next step to predict future velocity.

Compared with deep learning models such as LSTM, NARX has lower computational complexity, making it suitable for real-time online training and prediction. During periods of normal DVL operation, NARX can be trained alongside the navigation system; when DVL signals are interrupted, it can immediately switch to closed-loop mode for multi-step prediction. Even for several hundred future observation epochs, the computational load remains small, meeting the strict real-time requirements of underwater navigation systems.

This section introduces the basic principles of NARX. The next section will detail how the velocity prediction model is constructed based on NARX.

### 3.2. Construction of the Velocity Prediction Model

Based on the NARX principles introduced above, this study constructs a velocity prediction model for AUVs using actual navigation data. The model is trained to predict the vehicle velocity, which serves as the system output and can be expressed as:

(15) $$V=\left[\begin{array}{cccc} v_{k}, & v_{k-1}, & \ldots & v_{k-d_{v}} \end{array}\right]$$

The exogenous input data $u_{k}$ include velocity increments $\Delta v_{k,k+1}$ from the navigation system, attitude information $\varphi_{k}^{n}$ provided by the SINS (roll, pitch, and yaw), and velocity derived from propeller speed $V_{RPM}^{k}$.

(16) $$U=[\begin{array}{cccc} u_{k}, & u_{k-1}, & \ldots & u_{k-d_{u}} \end{array}]$$

(17) $$u_{k}=[\begin{array}{ccc} V_{RPM}^{k}, & \Delta v_{k,k+1}, & \varphi_{k}^{n} \end{array}]$$

Here, the parameter $V_{RPM}^{k}$ represents the conversion coefficient between the propeller speed measured by the RPM sensor and the actual forward velocity in the body frame observed by DVL during stable cruising. It can be expressed as:

(18) $$V_{RPM}^{k}=\sum_{i=1}^{N}\frac{V_{b}^{i}}{n_{i}}\times n_{k}$$

where n denotes the raw propeller speed measured by the RPM sensor.

The reasons for selecting these exogenous input parameters are as follows. First, as primary external inputs, they provide absolute observational information, offering reliable references for the NARX prediction model, effectively suppressing error accumulation and preventing divergence in state estimation. In this study, $V_{RPM}^{k}$ is used as a key exogenous input to provide absolute propulsion information for the AUV. However, during maneuvers, the propeller speed may experience delays or transient inconsistencies, and alone cannot fully reflect the actual velocity. To improve prediction accuracy, the propeller speed is combined with velocity increments from the navigation system and attitude information from the SINS, allowing the model to more accurately capture the dynamic response of the AUV.

Among these inputs, the velocity increments $\Delta v_{k,k+1}$ reflect the instantaneous acceleration and deceleration of the AUV, capturing short-term dynamic variations and enhancing the model’s sensitivity to velocity changes. The SINS-provided attitude information (roll, pitch, and heading) captures the coupling effects of heading and attitude adjustments on velocity. By combining these exogenous inputs with the historical output sequences, the NARX model can effectively learn the dynamic characteristics of the AUV, achieving accurate prediction of both the current and short-term future velocities.

The NARX model is trained exclusively on data collected during normal DVL operation. Data from DVL outage periods are used only for testing and are not included in training. In the experiments, the complete trajectory is divided chronologically into a training set and a test set. These two sets are temporally independent, ensuring no data leakage between them. Hyperparameters are selected via grid search, with the objective of minimizing the mean squared error on the validation set. The final configuration is input delay 4, feedback delay 2, and hidden layer size 10. The NARX model is trained offline and deployed with fixed parameters. The inference time per step is short and fully meets the real-time requirements of the navigation system. Moreover, its lightweight structure enables efficient inference without relying on GPU acceleration. The detailed parameter settings of the model are summarized in Table 3.

## 4. Simulation and Experiments

### 4.1. Experimental Setup

To verify the effectiveness of the proposed method, both simulation and real AUV trajectory data are used. First, to evaluate the performance of the AHR-EKF method in three-dimensional directions, it is compared with EKF, HR-EKF, and A-EKF under the SINS/DVL integrated navigation framework. On this basis, a PS is introduced to form the PS-AHR-EKF method. A rigorous ablation study is conducted. All methods are evaluated using the same AUV trajectory under identical conditions to ensure a fair comparison of navigation accuracy.

Second, relying on the SINS/DVL/PS integrated AHR-EKF algorithm, additional experiments are further designed to verify the effectiveness of the constructed NARX-based velocity prediction model in correcting the RPM-based velocity measurement results during DVL outages. Three comparison cases are set up, including no-velocity processing during DVL outages (Without-DVL), the velocity in the carrier coordinate system directly converted from the propeller RPM (V-RPM), and the NARX-based velocity prediction model constructed in this paper (V-NARX). Through comparative experiments of simulation and sea trial, the practical effect and advantages of the proposed method in actual underwater navigation scenarios are verified.

The following sections present a detailed analysis and discussion of the above experiments.

### 4.2. Simulation Experiments

**Figure 6:**
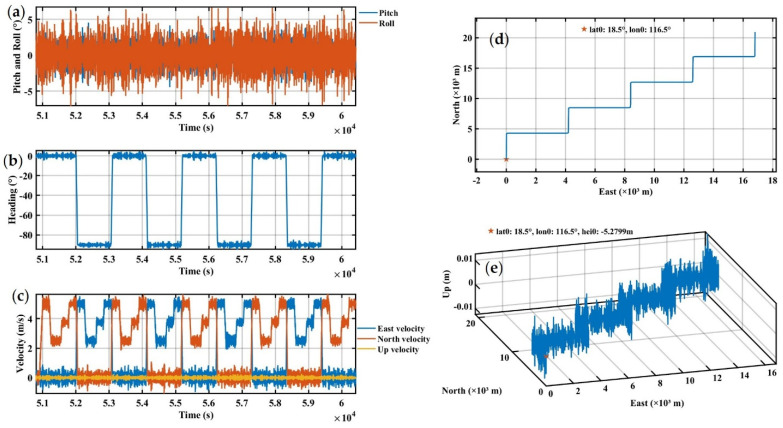
Attitude, velocity, and trajectory data during the simulated AUV trial. (**a**) Attitude angles (pitch, roll); (**b**) attitude angles (heading); (**c**) navigation velocities; (**d**) 2D horizontal trajectory; and (**e**) 3D spatial trajectory.

As shown in Figure 6, a simulated AUV trajectory with a length of approximately 38,000 m and a duration of 9625 s is designed. The trajectory includes multiple turning maneuvers, and the velocity varies continuously during the motion.

Based on this trajectory, IMU, DVL, and PS data are simulated. The sensor error parameters are set according to real devices, and the detailed error and noise parameters are listed in Table 4. The sampling frequencies of the IMU, DVL, and PS are 100 Hz, 1 Hz, and 1 Hz, respectively.

To independently validate the effectiveness of the proposed AHR-EKF algorithm in three dimensions and to accurately evaluate the contribution of the pressure sensor to the vertical channel, a hierarchical validation strategy is adopted in this study. First, the positioning accuracy of four methods—EKF, HR-EKF, A-EKF, and AHR-EKF—is compared under the SINS/DVL integration framework. This comparison is designed to evaluate the estimation performance of the AHR-EKF in three dimensions using only velocity observations, thereby excluding the interference of the direct depth constraint from the pressure sensor on the vertical channel. On this basis, the pressure sensor is further introduced to form the full SINS/DVL/PS integrated navigation scheme (i.e., PS-AHR-EKF), demonstrating the overall system performance and the constraint effect of the pressure sensor in the vertical direction. It should be noted that the validation of AHR-EKF under the SINS/DVL framework serves only to demonstrate the effectiveness of the robust adaptive filtering algorithm itself. The complete navigation scheme ultimately proposed in this paper is the integrated application of AHR-EKF under the SINS/DVL/PS three-sensor configuration.

**Figure 7:**
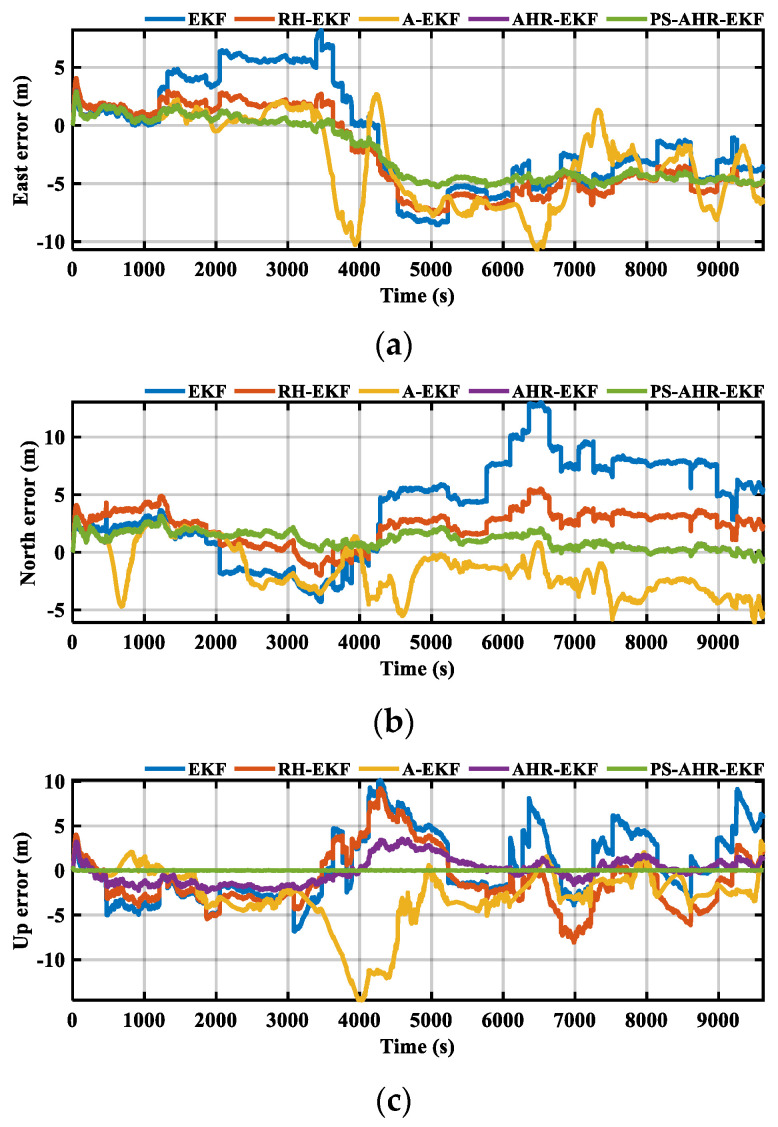
Positioning error comparison of different filtering algorithms under the simulation trajectory with DVL available, computed against the reference trajectory. (**a**) East errors; (**b**) north errors; and (**c**) up errors.

As shown in Figure 7 and Table 5, due to the combined effects of sensor noise and abnormal measurements, the conventional EKF cannot effectively identify or suppress outliers. This leads to poor filter stability and large position errors. Based on this, the HR-EKF employs the Huber robust function to detect and reject large outliers, and to adjust the error covariance accordingly. However, it has limited adaptability to small disturbances and time-varying noise. The A-EKF introduces the Sage–Husa adaptive mechanism into the EKF framework. It can estimate noise statistics online and adjust measurement weights in real time, making it more sensitive to time-varying noise and weak outliers. However, its ability to suppress strong outliers is limited. The correction may also propagate to subsequent epochs, which can cause imbalance in measurement weighting and degrade navigation accuracy.

In contrast, the AHR-EKF combines the robustness of the Huber method with the adaptability of the Sage–Husa approach. It can suppress strong outliers and adapt to time-varying noise effectively, resulting in improved overall filtering performance. Compared with HR-EKF, the AHR-EKF improves position accuracy by 19.70%, 52.35%, and 58.89% in the east, north, and up directions, respectively. Compared with A-EKF, the improvements are 26.35%, 50.44%, and 67.66%. These results demonstrate that the AHR-EKF provides better robustness against abnormal measurements, better adaptation to time-varying noise, and more stable navigation accuracy in complex underwater environments.

The PS has the advantages of high reliability, low cost, and continuous output. It provides strong depth constraints and is effective in suppressing vertical error divergence. As shown in Figure 7c and the corresponding Table 5, the PS-AHR-EKF significantly reduces vertical position errors after introducing depth observations, while maintaining the same optimal accuracy in the horizontal directions.

**Figure 8:**
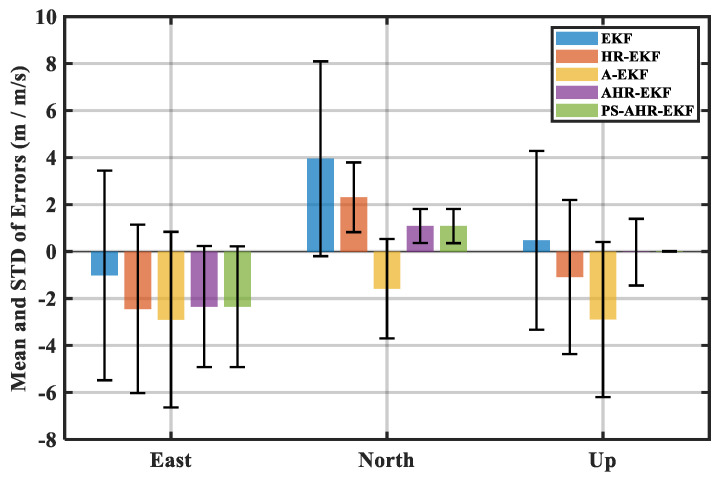
Mean and standard deviation of position errors for different filtering algorithms in three directions under the simulation trajectory with DVL available, computed against the reference trajectory.

As shown in Figure 8, the mean and standard deviation of velocity errors in the east, north, and up directions are compared for different filtering algorithms. The results indicate that, under the SINS/DVL integrated navigation framework, the AHR-EKF achieves the lowest error mean in all three directions and significantly reduces the standard deviation, demonstrating superior overall performance.

Furthermore, after introducing PS-based depth constraints, the PS-AHR-EKF effectively suppresses errors in the vertical channel. At the same time, it does not degrade the accuracy or stability in the horizontal directions. This confirms the important role of depth constraints in preventing vertical error divergence.

To address the degradation of navigation accuracy during DVL outages, a NARX-based velocity prediction model was introduced on top of the AHR-EKF framework. To evaluate its performance, DVL measurements were intentionally disabled between 7400 s and 9625 s in the simulated trajectory, simulating a bottom-tracking failure scenario. Only PS data and SINS observations were retained.

**Figure 9:**
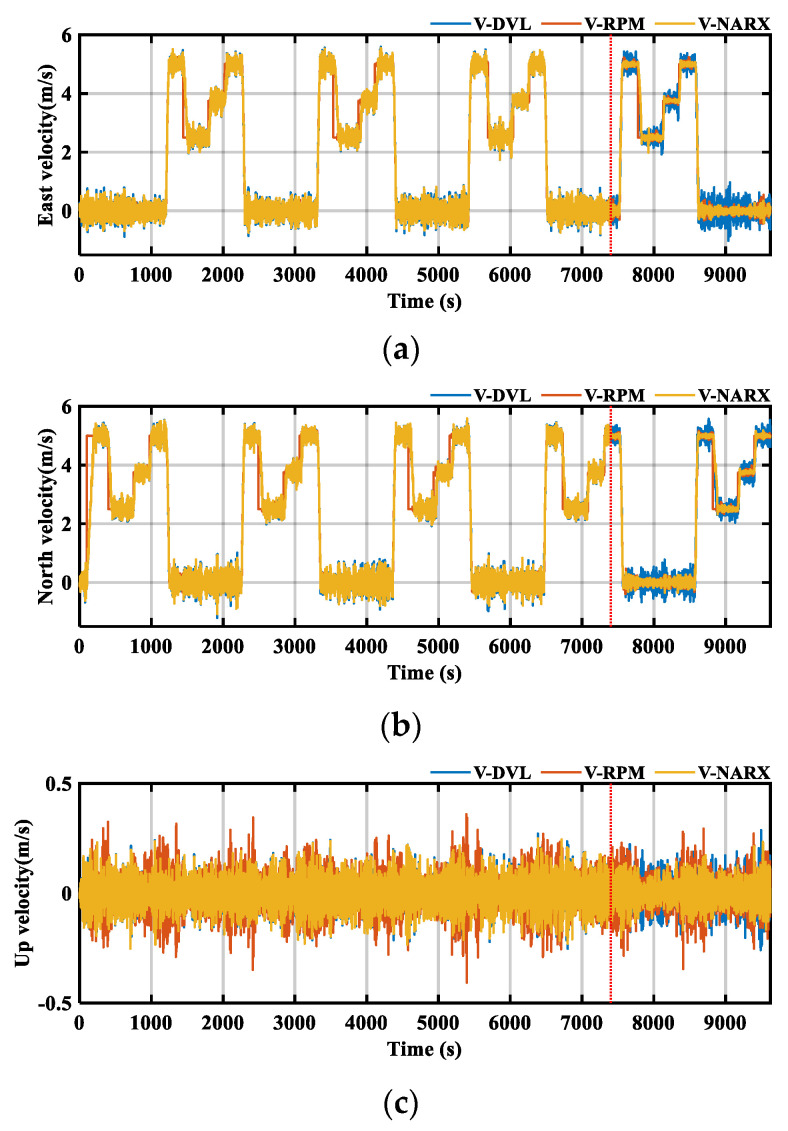
Comparison of velocity estimation results between V-DVL, V-RPM, and V-NARX under simulated DVL outage conditions, computed against DVL reference velocity. (**a**) East velocity; (**b**) north velocity; (**c**) and up velocity.

As shown in Figure 9, three velocity signals were compared: the DVL-measured velocity, the velocity directly inferred from the RPM, which is also an exogenous input to the NARX velocity prediction model, and the velocity predicted by the proposed NARX-based model. Due to propeller response delays and hydrodynamic disturbances, the V-RPM signal exhibits amplitude bias and phase lag during AUV maneuvers. In contrast, the V-NARX model effectively captures both the overall trend and short-term fluctuations of the AUV velocity, showing high consistency with the DVL measurements in all three directions. According to Table 6, the RMS errors of V-NARX in the east, north, and up directions are 0.264 m/s, 0.229 m/s, and 0.093 m/s, respectively. Compared with the V-RPM method, which has RMS errors of 0.328 m/s, 0.339 m/s, and 0.111 m/s, the errors are reduced by approximately 19.5%, 32.5%, and 16.2%. These results indicate that V-NARX effectively compensates for the response lag and environmental disturbances in propeller-speed-based velocity estimation, providing a more accurate and reliable velocity input for the integrated navigation system.

**Figure 10:**
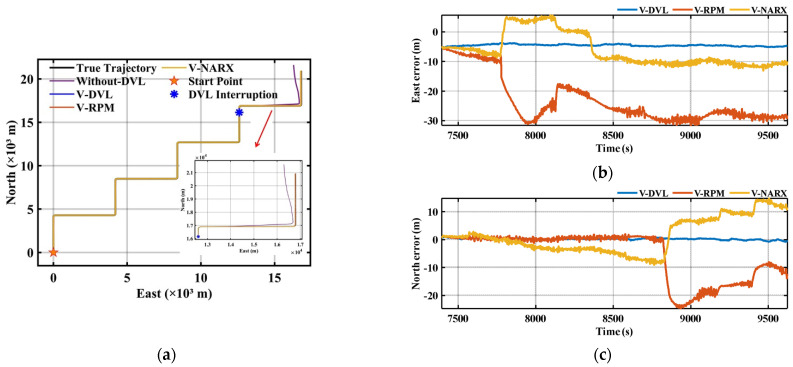
Trajectory and position error comparison of different methods under simulated DVL outages, computed against the reference trajectory. (**a**) Horizontal trajectory; (**b**) east errors; (**c**) and north errors.

To further evaluate the performance of the AHR-EKF method with NARX-based velocity prediction under DVL outages, three schemes were compared: pure INS, V-RPM, and V-NARX. As shown in Figure 10, without any velocity constraints, the pure INS solution diverges rapidly, and the estimated trajectory deviates significantly from the true path. The V-RPM scheme can partially suppress the errors but is still affected by system bias and response lag. In contrast, the proposed NARX-based velocity prediction effectively compensates for conversion errors, showing significant improvement.

As indicated in Table 7, in the Without-DVL scenario, errors accumulate rapidly, with RMS values reaching 234.400 m and 321.775 m in the east and north directions, respectively. This shows that navigation accuracy cannot be guaranteed by inertial navigation alone in the absence of external velocity observations. The V-RPM strategy lowers the root mean square errors to 24.699 m in the east direction and 10.218 m in the north direction, which can keep the basic movement tendency yet still produce obvious positioning deviations. In contrast, the proposed V-NARX method achieves remarkable performance gains. Its root mean square error reaches 8.397 m in the east direction and 6.530 m in the north direction, bringing about accuracy improvements of around 66.0% in the east direction and 36.1% in the north direction. It should be noted that, in the closed-loop prediction mode, the NARX model uses each predicted value as the input for the next step. Consequently, the prediction error accumulates gradually as the prediction horizon increases.

These results indicate that the NARX-based velocity prediction method preserves trajectory continuity and suppresses rapid error accumulation during DVL outages, providing more reliable velocity constraints for the integrated navigation system in emergency scenarios.

### 4.3. Sea Trial Experiment

**Figure 11:**
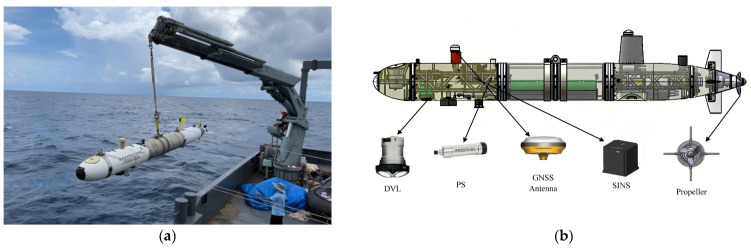
The AUV used in the sea trial and its navigation sensor configuration. (**a**) AUV deployment at sea; (**b**) sensor layout and key components.

To further verify the engineering applicability of the proposed AHR-EKF method with RPM constraints in real underwater environments, a sea trial experiment was conducted. As shown in Figure 11, the experimental data were collected using an AUV platform in a sea area of China in August 2023. The onboard sensors included SINS, DVL, PS, and an RPM acquisition module. The fiber-optic SINS employed in this study was the Conch 98FS, developed by CSSC Navigation Technology Co., Ltd. (Beijing, China), primarily intended for shipboard applications. The DVL utilized was a 300 kHz self-contained acoustic Doppler current profiler (ADCP) developed by the Institute of Acoustics, Chinese Academy of Sciences (Beijing, China). The PS used was the ISD4000, manufactured by Impact Subsea Ltd. (Aberdeen, Scotland, UK). The update rates of the IMU, DVL, and PS were 100 Hz, 1 Hz, and 1 Hz, respectively. The detailed sensor accuracy parameters are listed in Table 8. In this experiment, the Veripos high-precision differential GNSS service was employed as the position reference benchmark. This system utilizes multi-frequency, multi-constellation PPP technology, enabling horizontal positioning accuracy better than 10 cm under dynamic conditions. All GNSS measurements were time-synchronized and transformed to the local navigation coordinate frame, providing the ground-truth reference for accuracy evaluation of the navigation methods.

**Figure 12:**
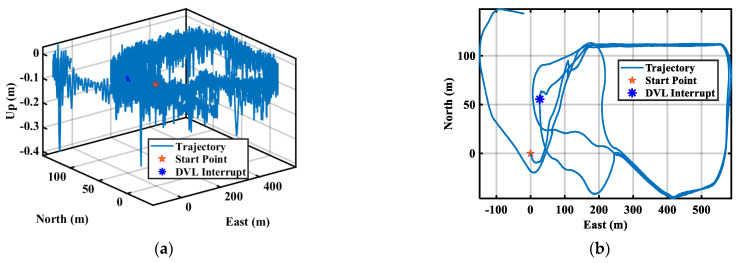
3D and 2D navigation trajectories during the sea trial. (**a**) 3D spatial trajectory; (**b**) 2D horizontal trajectory.

The actual trajectory of the AUV is shown in Figure 12. The total duration of the trajectory is 4788 s, covering typical operating conditions such as straight-line navigation, turning maneuvers, and variable-speed motion. To comprehensively evaluate the robustness of the proposed algorithm against abnormal measurements and DVL outages, several abnormal measurement epochs were added to the original data. In addition, DVL measurements were intentionally interrupted at 3500 s to simulate a bottom-tracking failure scenario. During this period, only the PS and RPM information were used as auxiliary constraints. The three-dimensional trajectory clearly shows the motion path and depth variation in the AUV in a real sea environment, providing a realistic and complete experimental basis for subsequent navigation performance evaluation.

**Figure 13:**
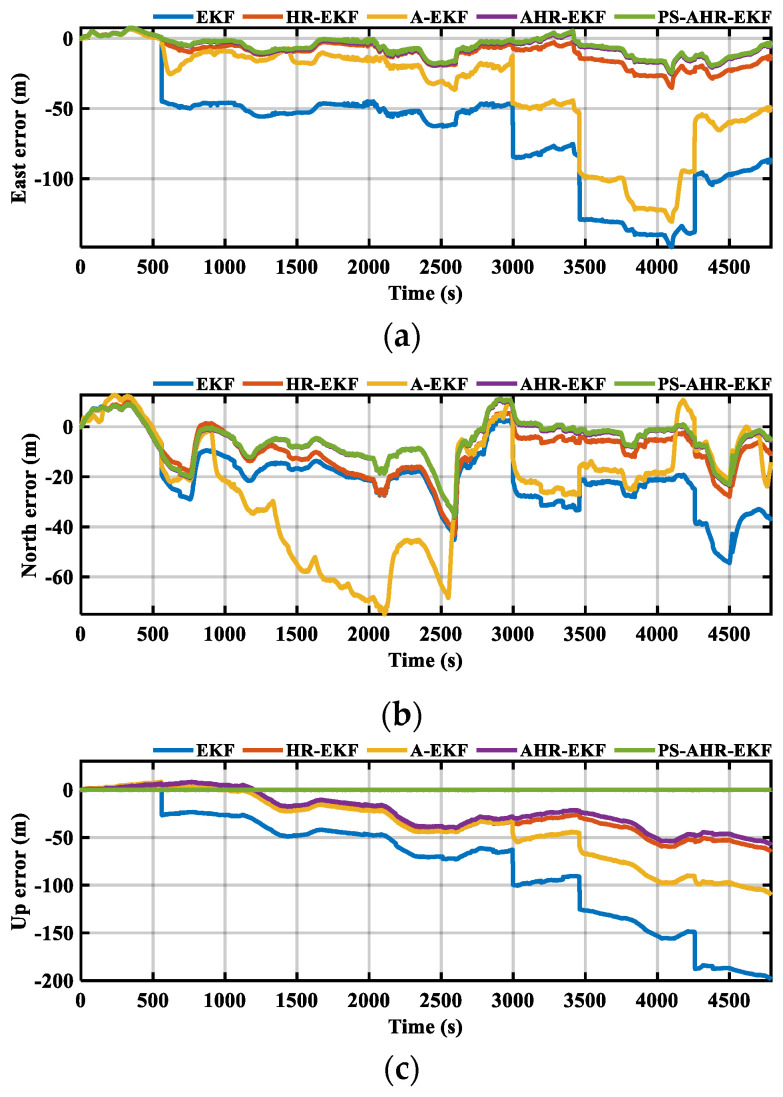
Positioning error comparison of different filtering algorithms under the sea trial trajectory with DVL available, computed against the GNSS reference trajectory. (**a**) East errors; (**b**) north errors; and (**c**) up errors.

The validation strategy for the sea trial is consistent with that used in the simulation. As shown in Figure 13 and Table 9, under the SINS/DVL framework, the conventional EKF is sensitive to noise and abnormal measurements. Its RMS position errors in the east, north, and up directions reach 77.839 m, 23.777 m, and 97.308 m, respectively. Such accuracy and stability cannot meet the requirements of long-duration AUV operations.

The HR-EKF can identify and suppress large abnormal measurements, significantly reducing both horizontal and vertical errors compared with the EKF. However, this method focuses mainly on outlier rejection, and its filtering accuracy can still be improved. The A-EKF can estimate noise statistics online and adjust measurement weights dynamically. It shows some adaptability to weak disturbances and time-varying noise. Nevertheless, it lacks sufficient robustness against strong outliers, and the corrections may cause weight imbalances in subsequent epochs. As a result, its overall performance is worse than that of the HR-EKF.

The AHR-EKF combines the robustness of Huber estimation with the adaptability of Sage–Husa filtering. It provides both outlier suppression and noise adaptation, and its filtering performance is superior to methods that use only one of these mechanisms. Compared with the HR-EKF, the AHR-EKF improves position accuracy by 31.04%, 24.68%, and 12.77% in the east, north, and up directions, respectively. Compared with the A-EKF, the improvements are 83.78%, 69.89%, and 45.24%. These results demonstrate the superiority of the adaptive robust fusion framework in complex underwater environments.

After introducing the PS into the AHR-EKF, under the full SINS/DVL/PS integration, the PS-AHR-EKF reduces the up-direction RMS position error from 29.217 m to 0.061 m. This reflects the direct depth constraint provided by the pressure sensor in the up direction, without degrading horizontal positioning performance. The significant improvement in vertical accuracy is mainly attributed to the hard constraint imposed by the pressure sensor as an independent depth observation. These results indicate that the proposed SINS/DVL/PS-based AHR-EKF method has good applicability in complex deep sea environments and can provide effective technical support for long-duration autonomous AUV navigation.

**Figure 14:**
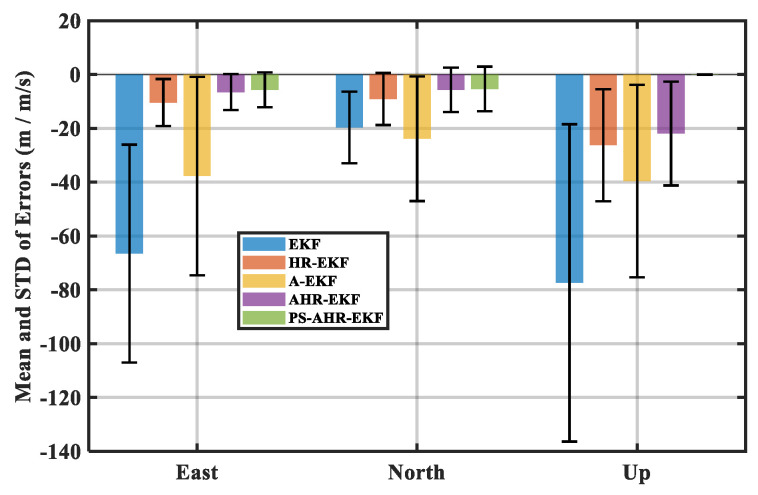
Mean and standard deviation of position errors for different filtering algorithms under sea trial DVL outages, computed against the GNSS reference trajectory.

As shown in Figure 14, the statistical comparison of error mean and standard deviation further indicates that HR-EKF and A-EKF reduce the error mean to some extent, and their biases are significantly smaller than those of the conventional EKF. By integrating robust estimation and adaptive estimation, the AHR-EKF effectively controls the error mean and achieves the best overall performance.

**Figure 15:**
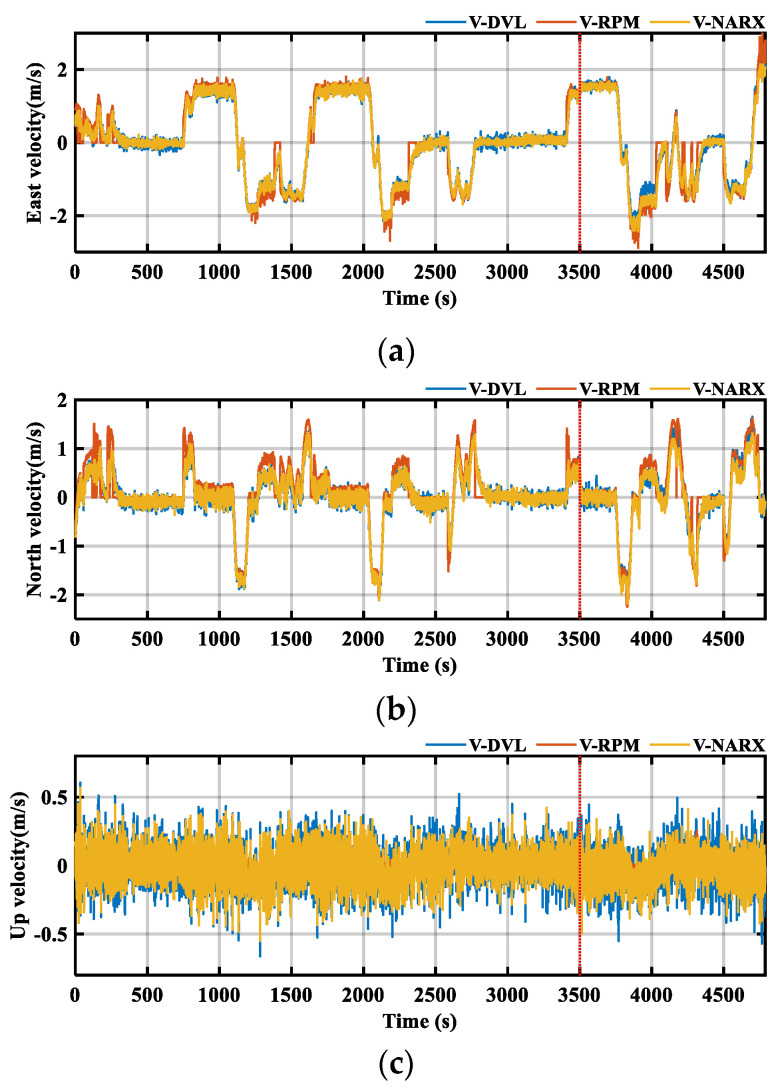
Velocity estimation results of V-RPM and V-NARX under sea trial DVL outages, computed against V-DVL reference velocity. (**a**) East velocity; (**b**) north velocity; (**c**) and up velocity.

As shown in Figure 15, the V-RPM method exhibits significant deviations from the DVL reference velocity, especially during acceleration, deceleration, and rapid velocity transitions. In contrast, the proposed V-NARX model provides a much better fit to the actual AUV velocity. The results show that the model not only captures short-term velocity fluctuations but also reflects the overall velocity trend, demonstrating its ability to model the nonlinear dynamics of the AUV propulsion system and maneuvering behavior.

According to Table 10, the RMS errors of the V-NARX model in the east and north directions are 0.166 m/s and 0.148 m/s, respectively, compared with 0.315 m/s and 0.225 m/s for the V-RPM method. This corresponds to reductions of approximately 47.2% and 34.2%.

The results indicate that V-NARX effectively compensates for modeling errors caused by nonlinear propulsion characteristics, hydrodynamic effects, and environmental disturbances.

It is worth noting that the sea trial trajectory covered typical operating conditions, including straight-line navigation, turning maneuvers, and variable-speed motion, with the AUV heading changing continuously throughout the entire voyage. In real ocean environments, different headings correspond to different current velocities, which in turn cause variations in propeller efficiency. Despite these dynamic changes, the proposed V-NARX model maintained stable and accurate velocity predictions. These results fully demonstrate the model’s generalization capability under varying sea state conditions. They also indirectly confirm the model’s robustness to dynamic changes in propulsion efficiency and external disturbances, as long as such variations remain within the dynamic range covered by the training data.

**Figure 16:**
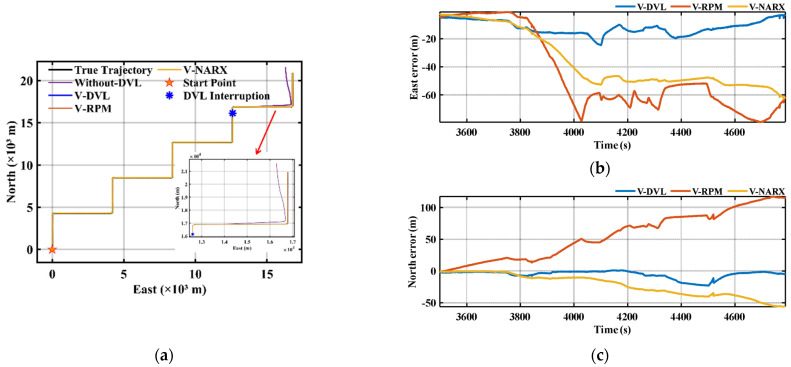
Trajectory and position error comparison of different methods under sea trial DVL outages, computed against the GNSS reference trajectory. (**a**) Horizontal trajectory; (**b**) east errors; and (**c**) north errors.

To evaluate the emergency navigation performance of the proposed V-NARX method under DVL outages, a comparative experiment was conducted during the DVL failure period from 3500 s to 4788 s. The velocity and trajectory results are shown in Figure 16.

Figure 16a compares the horizontal trajectories of different methods during the DVL outage. In the Without-DVL scenario, the INS trajectory quickly diverges from the reference trajectory and fails to reflect the actual AUV motion. The V-RPM method can maintain the general motion trend, but its trajectory shows systematic deviations due to ocean current disturbances and variations in propulsion efficiency. In contrast, the V-NARX trajectory agrees well with the reference, confirming its effectiveness in compensating for propeller speed conversion errors.

Figure 16b,c show the east and north positioning error evolution over time. At the early stage of the DVL outage, the errors of the three methods are similar. As time progresses, the Without-DVL errors grow in an approximately quadratic manner, consistent with the theoretical error growth caused by accelerometer bias integration.

According to Table 11, the V-RPM method yields final RMS errors of 52.820 m and 67.057 m, while the V-NARX method reduces them to 41.160 m and 28.023 m, corresponding to reductions of 22.1% and 58.2%.

It should be noted that although the V-NARX method significantly outperforms the V-RPM method, its positioning accuracy still does not reach the level achieved with V-DVL operation. The value of this method lies primarily in providing effective velocity constraints during DVL outages to suppress rapid INS error divergence, rather than completely replacing DVL velocity measurements.

## 5. Conclusions

This paper proposes an integrated navigation approach for AUVs combining the AHR-EKF with NARX-based velocity prediction to address challenges such as time-varying noise, abnormal measurements, and DVL outages in complex underwater environments. The main conclusions are summarized as follows:

(1) AHR-EKF method: The proposed approach integrates Huber robust estimation and Sage–Husa adaptive filtering within the EKF framework. It can suppress abnormal measurements and adapt to time-varying noise simultaneously. Simulation and field-test results show that AHR-EKF achieves higher position accuracy than EKF, HR-EKF, and A-EKF, with improved robustness and adaptability.
(2) NARX-based velocity prediction: During DVL outages, the proposed NARX model predicts short-term AUV velocity using propeller speed, velocity increments from the navigation system, and SINS attitude information as exogenous inputs. Compared with V-RPM, V-NARX reduces RMS errors by 47.2% in the east direction and 34.2% in the north direction, closely tracking DVL measurements. This preserves trajectory continuity and effectively suppresses rapid INS error accumulation.
(3) Navigation performance under DVL outages: When DVL measurements are unavailable, the proposed AHR-EKF with V-NARX maintains stable navigation. Compared with V-RPM, the method reduces RMS errors by approximately 22.1% in the east direction and 58.2% in the north direction. It significantly outperforms pure INS and V-RPM schemes and extends the reliable navigation duration without DVL support.

In summary, the proposed method achieves satisfactory accuracy and robustness in complex underwater environments, demonstrating promising engineering application potential and providing a useful reference for long-duration AUV operations. Nevertheless, certain limitations remain. The NARX model is trained offline with fixed-parameter deployment and does not support real-time online updating. When the propulsion system undergoes fundamental changes, retraining between missions is required. In addition, the DVL outages in this study are artificially induced, which may differ from naturally occurring failures in real ocean environments. The model accuracy also depends on the reliability of the propeller-speed-to-velocity mapping. Furthermore, the validation is based on a limited set of trajectories and operating conditions, without covering extreme environments such as deep sea or under-ice scenarios. To address these limitations, future work will conduct validation experiments under naturally occurring DVL outage conditions, investigate the impact of propulsion system variations on model accuracy, and explore online sequential learning, domain adaptation, and meta-learning strategies to enable rapid and low-cost adaptation to changing operating conditions across diverse AUV platforms and marine environments.

## Figures and Tables

**Table 1:** Comparison of existing methods and positioning of this work.

Method Category	Core Strategy	Limitation/Advantage
Huber robust filtering	Suppresses outliers via weight adjustment	Limited adaptability to time-varying noise
Sage–Husa adaptive filtering	Estimates noise covariance online	Sensitive to large outliers; risk of covariance non-positive definiteness
ML-based DVL compensation	Predicts velocity using data-driven models	Ignores abnormal measurements and noise variations during DVL outages; high computational cost and insufficient real-time performance
AHR-EKF and NARX	Jointly handles outliers, noise variation, and DVL outages via conditional triggering and prediction–filtering collaboration	Lightweight, suitable for embedded deployment; jointly handles outliers, time-varying noise, and DVL outages

**Table 2:** Algorithm flow of the AHR-EKF.

AHR-EKF Algorithm Flow
**Inputs:** The initial state ${\hat{X}}_{0}$, initial covariance ${\hat{P}}_{0}$, measurement ${\hat{P}}_{0}$, state transition matrix $\Phi_{k/k-1}$, measurement matrix $H_{k}$, initial process noise covariance $Q_{0}=\mathrm{diag}\left(\sigma_{arw}^{2},\sigma_{arw}^{2},\sigma_{arw}^{2},\sigma_{vrw}^{2},\sigma_{vrw}^{2},\sigma_{vrw}^{2}0,0,0,\sigma_{gb}^{2},\sigma_{gb}^{2},\sigma_{gb}^{2},\sigma_{ga}^{2},\sigma_{ga}^{2},\sigma_{ga}^{2}\right)$. $\sigma_{arw}$,$\sigma_{vrw}$, $\sigma_{gb}$ and $\sigma_{ga}$ denote the variances of angular random walk, velocity random walk, gyro bias, and accelerometer bias, respectively. The numerical values of these parameters are all known. The initial measurement noise covariance is $R_{0}=0.001\cdot I_{5\times 5}$. **Step 1:** Initialize the filter parameters, including the Huber threshold λ, the outlier detection threshold P, the forgetting factor b, and the upper and lower bounds of the measurement noise covariance. It should be noted that the following parameter values are adopted in this paper: λ=5.0, P=0.95 and b=0.99. The measurement noise covariance matrix ${\bar{R}}_{k}$ is updated in diagonal form and is constrained within the bounds $R_{k}^{\min}=0.02\cdot R_{0}$ and $R_{k}^{\max}=50\cdot R_{0}$. **Step 2:** Perform state prediction to obtain the predicted state and covariance: ${\hat{X}}_{k}^{-}=\Phi_{k/k-1}{\hat{X}}_{k-1}$, $P_{k}^{-}=\Phi_{k/k-1}{\hat{P}}_{k-1}\Phi_{k/k-1}^{T}+Q_{k-1}$. **Step 3:** Compute the innovation $V_{k}$ and the normalized residual $u_{k}$: $V_{k}=Z_{k}-H_{k}{\hat{X}}_{k}^{-}$, $u_{k}\frac{\left|V_{k}\right|}{\sqrt{\mathrm{diag}({\bar{R}}_{k})}}$. **Step 4:** Compute the Huber weights based on the normalized residuals: $p_{k}=\left\{\begin{array}{ll} 1, & u_{k}\leq \lambda \\ \lambda /u_{k}, & u_{k}>\lambda \end{array}\right.$ where $\;p_{k}$ is the Huber threshold. If the residual is large, the corresponding measurement weight is reduced, thereby weakening the influence of abnormal measurements on the filter result. **Step 5:** Determine whether the measurement is abnormal. If $p_{k}(j,j)<P$, the measurement is considered an outlier, and the Sage–Husa update is skipped; otherwise, proceed to the Sage–Husa adaptive update. **Step 6:** When the measurement is normal, estimate the measurement noise covariance using the Sage–Husa method: ${\tilde{R}}_{k}=V_{k}V_{k}^{T}-H_{k}P_{k}^{-}H_{k}^{T}$, and update it with the weighting coefficient $\beta_{k}$. **Step 7:** Compute the Kalman gain and perform the state update: $K_{k}=P_{k}^{-}H_{k}^{T}{\left(H_{k}P_{k}^{-}H_{k}^{T}+R_{k}\right)}^{-1}$, ${\hat{X}}_{k}={\hat{X}}_{k}^{-}+K_{k}V_{k}$, ${\hat{P}}_{k}=(I-K_{k}H_{k})P_{k}^{-}$. **Output:** The state estimate ${\hat{X}}_{k}$ and the error covariance matrix ${\hat{P}}_{k}$ at the current time step.

**Table 3:** NARX neural network parameter settings for AUV velocity prediction.

Parameter	Settings
Input features	$\Delta v_{k,k+1}$ are the velocity increments from the navigation system, $\varphi_{k}^{n}$ is the attitude information from SINS, $V_{RPM}^{k}$ is the propeller speed-derived velocity
Target output	AUV velocity in the body frame, measured by DVL
Input/output processing	Remove constant rows and normalize to [−1, 1]
Input delays	4
Feedback delays	2
Hidden layer size	10
Training function	Levenberg–Marquardt
Performance function	MSE
Maximum epochs	1000
Closed-loop mode	Multi-step prediction using previous outputs as inputs

**Table 4:** Error parameter settings for the simulation trial.

Sensors	Error Value	Error Settings
IMU	Gyroscope Biases	0.01 deg/h
	Accelerometer Biases	100 ug
	Angular Random Walk	0.01 deg/sqrt(h)
	Velocity Random Walk	25 ug/sqrt(Hz)
	Data Rate	100 Hz
DVL	Scale Factor Error	0.5%
	Random Velocity Error	0.02 m/s
	Outlier Probability	0.004
	Maximum Outlier/Normal Noise	50
	Time-varying Noise	0.4 × sin(0.002t)
	Time Delay	5 ms
	Lever Arm	(0.2, −0.8, 0.5) m
	Data Rate	1 Hz
PS	Scale Factor Error	0.05%
	Random Depth Error	0.01 m
	Lever Arm	(0.5, 0.8, −0.5) m
	Data Rate	1 Hz

**Table 5:** Three-dimensional positioning RMSE comparison of different filtering algorithms under the simulation trajectory with DVL available, computed against the reference trajectory.

Method	RMS (m)		
	East	North	Up
EKF	4.578	5.727	3.832
HR-EKF	4.340	2.745	3.454
A-EKF	4.732	2.639	4.391
AHR-EKF	3.485	1.308	1.420
PS-AHR-EKF	3.482	1.308	0.021

**Table 6:** RMS velocity errors of V-RPM and V-NARX under simulated DVL outages, computed against V-DVL reference velocity.

Method	RMS (m/s)		
	East	North	Up
V-RPM	0.328	0.339	0.111
V-NARX	0.264	0.229	0.093

**Table 7:** RMS positioning errors of different methods (V-DVL, Without-DVL, V-RPM, and V-NARX) under simulated DVL outages, computed against the reference trajectory.

Method	RMS (m)	
	East	North
V-DVL	4.569	0.361
Without-DVL	234.400	321.775
V-RPM	24.699	10.218
V-NARX	8.397	6.530

**Table 8:** Main parameters and error specifications of the navigation sensors used in the sea trial.

Sensors	Error Value	Error Settings
IMU	Gyroscope biases	0.01 deg/h
	Accelerometer biases	3000 ug
	Angular random walk	0.01 deg/sqrt(h)
	Velocity random walk	300 ug/sqrt(Hz)
DVL	Maximum bottom tracking distance	230 m
	Bottom tracking accuracy	±0.4% ± 0.005 m/s
	Beam frequency	300,000 Hz
	Lever arm	(0; 0.61; 0.38) m
PS	Maximum depth	3000 m
	Accuracy	±0.01% full scale
	Resolution	0.001% full scale
	Lever arm	(0; −0.34; −0.07) m

**Table 9:** Three-dimensional positioning RMS errors of different filtering algorithms under the sea trial trajectory with DVL available, computed against the GNSS reference trajectory.

Method	RMS (m)		
	East	North	Up
EKF	77.839	23.777	97.308
HR-EKF	13.568	13.293	33.493
A-EKF	57.715	33.261	53.353
AHR-EKF	9.357	10.013	29.217
PS-AHR-EKF	8.628	9.874	0.061

**Table 10:** RMS velocity errors of V-RPM and V-NARX under sea trial DVL outages, computed against V-DVL reference velocity.

Method	RMS (m/s)		
	East	North	Up
V-RPM	0.315	0.225	0.141
V-NARX	0.166	0.148	0.135

**Table 11:** RMS positioning errors of different methods (Without-DVL, V-RPM, and V-NARX) under sea trial DVL outages, computed against the GNSS reference trajectory.

Method	RMS (m)	
	East	North
V-DVL	12.866	8.160
Without-DVL	33,094.377	26,099.902
V-RPM	52.820	67.057
V-NARX	41.160	28.023

## Data Availability

The data presented in this study are available on request from the corresponding author.
